# Feedforward inhibition ahead of ictal wavefronts is provided by both parvalbumin‐ and somatostatin‐expressing interneurons

**DOI:** 10.1113/JP277749

**Published:** 2019-03-18

**Authors:** R. Ryley Parrish, Neela K. Codadu, Connie Mackenzie‐Gray Scott, Andrew J. Trevelyan

**Affiliations:** ^1^ Institute of Neuroscience Medical School Framlington Place Newcastle upon Tyne NE2 4HH UK

**Keywords:** Epilepsy, seizure, interneurons, Ca^2+^ imaging

## Abstract

**Key points:**

- There is a rapid interneuronal response to focal activity in cortex, which restrains laterally propagating activity, including spreading epileptiform activity.
- The interneuronal response involves intense activation of both parvalbumin‐ and somatostatin‐expressing interneurons.
- Interneuronal bursting is time‐locked to glutamatergic barrages in the pre‐ictal period.
- Ca^2+^ imaging using conditional expression of GCaMP6f provides an accurate readout of the evolving firing patterns in both types of interneuron.
- The activation profiles of the two interneuronal classes are temporally offset, with the parvalbumin population being activated first, and typically, at higher rates.

**Abstract:**

Previous work has described powerful restraints on laterally spreading activity in cortical networks, arising from a rapid feedforward interneuronal response to focal activity. This response is particularly prominent ahead of an ictal wavefront. Parvalbumin‐positive interneurons are considered to be critically involved in this feedforward inhibition, but it is not known what role, if any, is provided by somatostatin‐expressing interneurons, which target the distal dendrites of pyramidal cells. We used a combination of electrophysiology and cell class‐specific Ca^2+^ imaging in mouse brain slices bathed in 0 Mg^2+^ medium to characterize the activity profiles of pyramidal cells and parvalbumin‐ and somatostatin‐expressing interneurons during epileptiform activation. The GCaMP6f signal strongly correlates with the level of activity for both interneuronal classes. Both interneuronal classes participate in the feedfoward inhibition. This contrasts starkly with the pattern of pyramidal recruitment, which is greatly delayed. During these barrages, both sets of interneurons show intense bursting, at rates up to 300Hz, which is time‐locked to the glutamatergic barrages. The activity of parvalbumin‐expressing interneurons appears to peak early in the pre‐ictal period, and can display depolarizing block during the ictal event. In contrast, somatostatin‐expressing interneuronal activity peaks significantly later, and firing persists throughout the ictal events. Interictal events appear to be very similar to the pre‐ictal period, albeit with slightly lower firing rates. Thus, the inhibitory restraint arises from a coordinated pattern of activity in the two main classes of cortical interneurons.

## Key points

- There is a rapid interneuronal response to focal activity in cortex, which restrains laterally propagating activity, including spreading epileptiform activity.
- The interneuronal response involves intense activation of both parvalbumin‐ and somatostatin‐expressing interneurons.
- Interneuronal bursting is time‐locked to glutamatergic barrages in the pre‐ictal period.
- Ca^2+^ imaging using conditional expression of GCaMP6f provides an accurate readout of the evolving firing patterns in both types of interneuron.
- The activation profiles of the two interneuronal classes are temporally offset, with the parvalbumin population being activated first, and typically, at higher rates.

## Introduction

In recent years, a new perspective on the spatial structure of seizures has been derived from detailed studies of propagating epileptic events recorded in brain slices (Wong and Prince, 1990; Trevelyan *et al*. 2006; Trevelyan *et al*. 2007; Losi *et al*. 2010; Cammarota *et al*. 2013; Sessolo *et al*. 2015) and *in vivo*, in both animal models (Prince and Wilder, 1967; Dichter and Spencer, 1969; Schwartz and Bonhoeffer, 2001; Timofeev *et al*. 2002; Timofeev and Steriade, 2004; Toyoda *et al*. 2015) and during spontaneously occurring seizures in humans (Truccolo *et al*. 2011; Schevon *et al*. 2012; Weiss *et al*. 2013; Weiss *et al*. 2015). A key feature of this work has been to distinguish between two spatial patterns of activity: a core area, where all neurons are involved (although some might be pushed into depolarizing block; Ziburkus *et al*. 2006; Sessolo *et al*. 2015), and a second territory that is characterized by very large amplitude field potentials, but surprisingly low levels of activity, which we termed the ictal penumbra (Trevelyan and Schevon, 2013; Weiss *et al*. 2013). This latter area appears to be the manifestation of a rapidly activated inhibition. This inhibitory response is a concept with a long history, being identified in the first report of intracellular recordings of cortical neurons *in vivo* (Powell and Mountcastle, 1959), and some of the earliest *in vivo* studies of epileptic discharge (Prince and Wilder, 1967; Dichter and Spencer, 1969).

Latterly, our understanding of this response has been extended greatly from *in vitro* studies of propagating ictal activity (Trevelyan *et al*. 2006, 2007; Cammarota *et al*. 2013; Sessolo *et al*. 2015). The recruitment of new territory is driven by the glutamatergic output from neighbouring territories that have already been incorporated into the ictal event, but when this glutamatergic bombardment starts, there can be a surprising delay before pyramidal cells are recruited. This delay appears to arise primarily from feedforward inhibition (Trevelyan *et al*. 2007). In one sense, this rapid interneuronal response could be argued to be the first recruitment to the ictal event, but we argue elsewhere (Trevelyan and Schevon, 2013; Trevelyan, 2016) that it is better to view this as the physiological response to the pathological ictal activity occurring in the adjacent territory, the final obstacle opposing the pathological spread. The critical pathological transition is in fact the recruitment of the pyramidal population, since these are the largest class of cortical neurons, and also the means by which seizures propagate further. Here we examine the pattern of interneuronal behaviour, immediately prior to the recruitment of the pyramidal cells.

There remains, however, uncertainty on which interneuronal populations are the primary source of this rapid inhibitory response to focal activation. Parvalbumin (PV)‐expressing interneurons are thought to play a prominent role, based both on theoretical grounds, because of their targeting the proximal dendrites and somata of pyramidal cells (Trevelyan and Watkinson, 2005; Pouille *et al*. 2013), and also on experimental observations of their activity patterns during epileptiform discharges (Cammarota *et al*. 2013; Sessolo *et al*. 2015; Parrish *et al*. 2018 *b*). Less clear is the role played by somatostatin (SST)‐expressing interneurons, which tend to target the more distal dendrites of pyramidal cells, and which, in passive models of dendritic integration might be expected to provide a weaker inhibitory effect (Jack *et al*. 1983). These distal dendrites, though, are themselves excitable; they have large numbers of voltage‐gated Ca^2+^ channels and NMDA receptors, which can sustain plateau potentials (Schiller *et al*. 2000) and dramatically increase the firing output of the neurons by triggering bursting behaviour (Larkum *et al*. 1999). Consistent with this, Lovett‐Barron *et al*. (2012) showed a surprisingly powerful effect of SST interneurons on suppressing pyramidal activity. The relative roles of these two interneuron populations is made more complicated by their mutual inhibition (and by other interneuron populations, such as VIP interneurons; Pfeffer *et al*. 2013; Karnani *et al*. 2016), and which may also vary in different brain regions (contrast Lovett‐Barron *et al*. 2012; Pfeffer *et al*. 2013). Interestingly, Pouille and Scanziani showed, in the CA1 region, that both populations contribute to feedforward inhibition, but are temporally offset from each other, with a transition from early PV activity to later SST activity, in response to physiological levels of glutamatergic drive (Pouille and Scanziani, 2004).

The role of different interneuron classes in epileptic events was examined recently *in vivo*, using both optogenetic and genetically expressed Ca^2+^ indicators (Khoshkhoo *et al*. 2017). Ictal events were triggered by repeated focal optogenetic activation of pyramidal cells – a kind of optogenetic kindling – and the interneuronal activity was visualized contralaterally using Ca^2+^ imaging, or suppressed using archaerhodopsin. The results were complex and quite difficult to interpret, in part because the imaging data and optogenetic control used wide‐field illumination, and so their precise spatial relationship to the electrophysiology signals was not known. An added complexity impacting on this study is that it is known that the neuropil signal during epileptiform discharges may distort the interpretation of Ca^2+^ signals with respect to neuronal firing (Trevelyan *et al*. 2006), and so it is necessary to distinguish between neuropil and somatic Ca^2+^ signals. To resolve these issues, we examined the activity patterns of three classes of cortical neuron, pyramidal cells and PV‐ and SST‐expressing interneurons, using cellular resolution imaging of Ca^2+^ signals in tandem with local patch clamp recordings of epileptiform discharges. We were able to derive an accurate read‐out of the pattern of firing in PV‐ and SST‐expressing interneurons, suggesting the presence of the Ca^2+^‐binding protein parvalbumin does not unduly distort the analysis of GCaMP6f imaging.

We use the 0 Mg^2+^ model in brain slices prepared from young adult mice. This has proved a very effective tool for studying the rapid interneuronal response (Trevelyan and Schevon, 2006), because its primary effect is to greatly enhance excitatory synaptic events by removing the voltage‐dependent block of NMDA receptors. As such, it creates regular surges of network activity with a large glutamatergic component (Trevelyan *et al*. 2006), allowing one to examine the rapid inhibitory response to these ‘network crises’. In contrast, another widely used pharmacological model, bathing slices in 4‐aminopyridine, appears to induce interneuronal bursts more directly, and can occur without any glutamatergic drive (Avoli and de Curtis, 2011), presumably because the baseline activity in interneurons is destabilized by the block of K^+^ conductance in these cells. It is important to appreciate this distinction in how interneuronal bursts arise in these two models.

We report that both PV and SST interneurons fire intensely during both the immediate pre‐ictal period (the term refers to the period just before the pyramidal recruitment) and also during interictal bursts, whereas pyramidal activity is strongly restrained despite the large glutamatergic drive at these times. We also report a transition from early PV activation to later SST activity, recapitulating the pattern identified previously for physiological drives (Pouille and Scanziani, 2004). We suggest that this is evidence of a coordinated interneuronal response that targets all excitable areas of pyramidal cells, in a way that optimizes the chance of restraining a pathological spreading wave of activity.

## Methods

### Ethical approval

All procedures performed were in accordance with the guidelines of the UK Home Office and the Animals (Scientific Procedures) Act 1986 and approved by the Newcastle University Animal Welfare and Ethical Review Body (AWERB ID no. 545). All authors understand the ethical guidelines under which *The Journal* operates and our work complies with the animal ethics checklist.

### Slice preparation

Male and female Emx1‐Cre (B6.129S2‐Emx1tm1(cre) Krj/J; Jackson Laboratory, Bar Harbor, ME, USA; stock no. 5628), PV‐Cre (B6; 129P2‐Pvalb<tm1(cre)Arbr>/J; Jackson Laboratory stock no. 8069), and SOM‐Cre (B6N.Cg.Ssttm2.1(cre)Zjh/J; Jackson Laboratory stock no. 18973) mice and C57/B6 mice (ages 3 – 12 weeks) were used in this study. Transgenic mice were back‐crossed with the C57/B6 line maintained at Newcastle University, and subsequently maintained on this C57/B6 background (Jackson Laboratory stock no. 000664). Mice were housed in individually ventilated cages in a 12 h light–12 h dark lighting regime. Animals received food and water *ad libitum*. Mice were sacrificed by cervical dislocation (Schedule 1) and brains were then removed and stored in ice cold cutting solution containing (in mm): 3 MgCl_2_; 126 NaCl; 26 NaHCO_3_; 3.5 KCl; 1.26 NaH_2_PO_4_; 10 glucose. Coronal sections of 350 μm were cut on a Leica VT1200 vibrating blade microtome (Leica Microsystem, Wetzlar, Germany). Slices were stored at room temperature, in a submerged holding chamber, for 1–4 h prior to experimentation. Solutions were bubbled with carboxygen (95% O_2_–5% CO_2_) in artificial cerebrospinal fluid (ACSF) containing (in mm): 2 CaCl_2_; 1 MgCl_2_; 126 NaCl; 26 NaHCO_3_; 3.5 KCl; 1.26 NaH_2_PO_4_; 10 glucose. The 0 Mg^2+^ solution is identical, except for omitting MgCl_2_.

### Dye loading

Slices were bulk loaded with Oregon Green 488 BAPTA‐1 (OGB1)‐AM ester as follows. OGB1‐AM (50μg vial, Thermo Fisher Scientific,Waltham, MA, USA) was mixed with 8 μl DMSO and 2 μl Pluronic F‐127 solution (10% in DMSO, Thermo Fisher Scientific). Meanwhile, the slices were preincubated at 37°C for 5 min in 3 ml ACSF with 8 μl Cremophor EL solution (0.5% in DMSO, Sigma‐Aldrich, Gillingham, UK). The OGB1–Pluronic F‐127–DMSO mixture was then added and the slices incubated for a further 30–40 min. The final concentrations were ∼12 μm OGB1‐AM ester, 0.6% DMSO, 0.002% Cremophor EL, 0.01% Pluronic F‐127. The slices were then placed back in ACSF for at least 30 min prior to transferring to the recording chamber.

In most experiments, once the tissue was in the recording chamber, it was bathed briefly in sulforhodamine (SR101, Thermo Fisher Scientific; 3 ml of 1 μm solution (in ACSF), and subsequently washed through for at least 15 min) to label the astrocyte population (Nimmerjahn *et al*. 2004). The SR101 was visualized using epifluorescence illumination, viewed through a standard rhodamine filter set (Olympus Corporation, Tokyo, Japan; U‐MRFPHQ filter block).

### Viral injections

PV‐Cre, SOM‐Cre, or Emx1‐Cre pups were injected with AAV9.Syn.flex.GCaMP6f or AAV5.Syn.Flex.tdTomato, purchased from the University of Pennsylvania vector core. Injections were performed either on the day of birth or on the following day. Pups were set on a stereotaxic frame and anaesthetized and maintained with 2% isoflurane, following application of EMLA cream (2.5% lidocaine and 2.5% prilocaine) to the left top of their head. Injections were made, using 10 μl Hamilton syringes with a bevelled 36‐gauge needle (World Precision Instruments, Sarasota, FL, USA), unilaterally into the lateral ventricle and overlying cortical plate, at about 1 mm anterior to lambda and 1 mm lateral to the midline into the left hemisphere, starting at 1.7 mm depth from the top of the dura mater, for a total of four separate 50 nl injections, deepest first and coming up 0.3 mm for each subsequent injection. Approximately 200 nl (∼1000 viral particles) were injected into the left hemisphere over a 10 min period. Pups were monitored until they awoke following the procedure and then returned to their home cage. These neonatal injections produced widespread cortical expression of GCaMP6f or tdTomato into the neurons of interest.

### Electrophysiology

Recordings were made using a Multiclamp 700B amplifier (Molecular Devices, San Jose, CA, USA) and pClamp software, digitized at 10 kHz. The bath was mounted on a Scientifica movable top plate (Scientifica, Uckfield, UK) fitted with a heater plate (Warner Instruments, Hamden, CT, USA), and the incoming solution (perfusion at 1–3 ml/min) was heated by a sleeve heater element (Warner Instruments). All imaging and electrophysiological recordings were done at 33–37°C. Whole‐cell patch‐clamp recordings were made using 3–7 MΩ pipettes (borosilicate glass; Harvard Apparatus, Camborne, UK) controlled with Patchmaster micromanipulators (Scientifica). Pipettes were filled with a KMeSO_4_‐based internal solution containing (in mm): 125 KMeSO_4_, 6 NaCl, 10 Hepes, 2.5 Mg‐ATP, 0.3 Na_2_‐GTP, 0.5% (w/v) biocytin, 5 mm QX‐314 (*N*‐(2,6‐dimethylphenylcarbamoylmethyl) triethylammoniumbromide), and 30 μm Alexa Fluor 568 or Alexa Fluor 594 (Thermo Fisher Scientific). For cell‐attached recordings, the electrode was filled with KMeSO_4_‐based internal solution without QX‐314. Interneurons were identified either using Ca^2+^ imaging or using tdTomato, exclusively expressed in either the PV or the SST cell population. There were no qualitative differences between the recordings from OGB1‐loaded and GcaMP6f‐expressing cells *versus* unloaded/non‐transfected slices. All experiments were run in ACSF without Mg^2+^ to induce epileptiform activity. The electrophysiological data were analysed off‐line using in‐house software implemented in MATLAB (The MathWorks, Natick, MA, USA) or Clampfit software (Molecular Devices). postsynaptic potentials (PSPs) were automatically identified by peaks in the derivative of the signal, and then individually confirmed or rejected, through a GUI implemented in MATLAB. PSP sampling included both baseline epochs and also the bursts of activity during interictal events, but not during full ictal events.

### Live tissue imaging

Live imaging experiments were performed using two different spinning disk confocal microscopes: (1) an Olympus DSU spinning disk BX/50WI upright microscope (UMPlanFL N ×20, 0.5 NA objective; Olympus), illuminated using a mercury arc lamp, controlled with a fast Sutter shutter (Sutter Instrument Co., Novato, CA, USA), using the standard Olympus FITC (U‐MGFPHQ) and rhodamine (U‐MRFPHQ) filter sets; and (2) a spinning disc confocal microscope (UMPlanFL N ×20, 0.5 NA objective). The tissue was illuminated with a 491 nm laser (Cobolt Calypso 50; Cobolt AB, Solna, Sweden) for visualization of the GCaMP6f, while the 561 nm laser (Cobolt Jive 50; Cobolt) was used to visualize the tdTomato and SR101 dyes. Both systems utilized Hamamatsu C9100 EM cameras (Hamamatsu Photonics (UK), Welwyn Garden City, UK) to collect images, run either by Simple PCI software (Digital Pixel, Brighton, UK; microscope 1) or VoxCell software (Visitech International, Sunderland, UK; microscope 2), both installed on Dell Precision computers (Dell Technologies, Round Rock, TX, USA).

Assessment of the GCaMP6f with respect to firing patterns was made by performing cell‐attached recordings of firing patterns in either PV‐expressing or SST‐expressing neurons that also conditionally expressed GCaMP6f. Concurrent imaging was performed at 10 Hz, and the number of action potentials was calculated for each imaging frame. Off‐line analysis of the images was performed using ImageJ (imagej.net, NIH, Bethesda, MD, USA) and in‐house software implemented on MATLAB. Having validated these analyses against the cell‐attached recordings of firing patterns, we then applied these also to assess imaging of populations of neurons during ictal events. In these experiments, we limited our analysis to neurons that were demonstrably healthy, in that they showed a prominent Ca^2+^ transient during the full ictal event. We aligned these recordings using the two most readily identifiable, features of the pyramidal *V*_clamp_ recordings, which was the start of the rhythmic (1–2 Hz) synaptic bombardments (*T*_i_) and the time of the peak inward current (*T*_e_) which our previous work has shown coincides closely with the main recruitment of the local pyramidal population.

### Statistics

Statistical analysis of Ca^2+^ network imaging and electrophysiology was performed using Prism (GraphPad Software, Inc., La Jolla, CA, USA) or MATLAB. Data were analysis with Student's *t* test or one‐way ANOVA with Tukey's test where appropriate. Non‐parametric data were analysed using the Wilcoxon rank sum test. Figures with electrophysiology traces were created in Igor Pro (WaveMetrics, Lake Oswego, OR, USA) and MATLAB.

## Results

### Pre‐ictal period is characterized by intense interneuronal activation

The recruitment of pyramidal neurons to a propagating ictal event, both *in vitro* (Trevelyan *et al*. 2006) and *in vivo* (Timofeev *et al*. 2002; Timofeev and Steriade, 2004), is characterized by a series of rhythmic depolarizations. The driving force for these depolarizations derives from temporally matched discharges in neighbouring territories (Trevelyan *et al*. 2006); the rhythmic glutamatergic drive at this time can be extremely large, and yet the firing is extremely restrained (Trevelyan *et al*. 2006; Trevelyan and Schevon, 2013). Ca^2+^ network imaging of the networks during this period of restraint shows a large neuropil signal, indicative of the synaptic bombardment, but the somatic signals, which provide a more accurate marker of neuronal firing, are greatly delayed in the majority of neurons. There are, however, small numbers of local neurons that appear to be activated from the start of the synaptic bombardment. The implication, from our previous work characterizing the late recruitment of pyramidal cells, is that the early recruits by contrast are interneurons.

**Figure 1:**
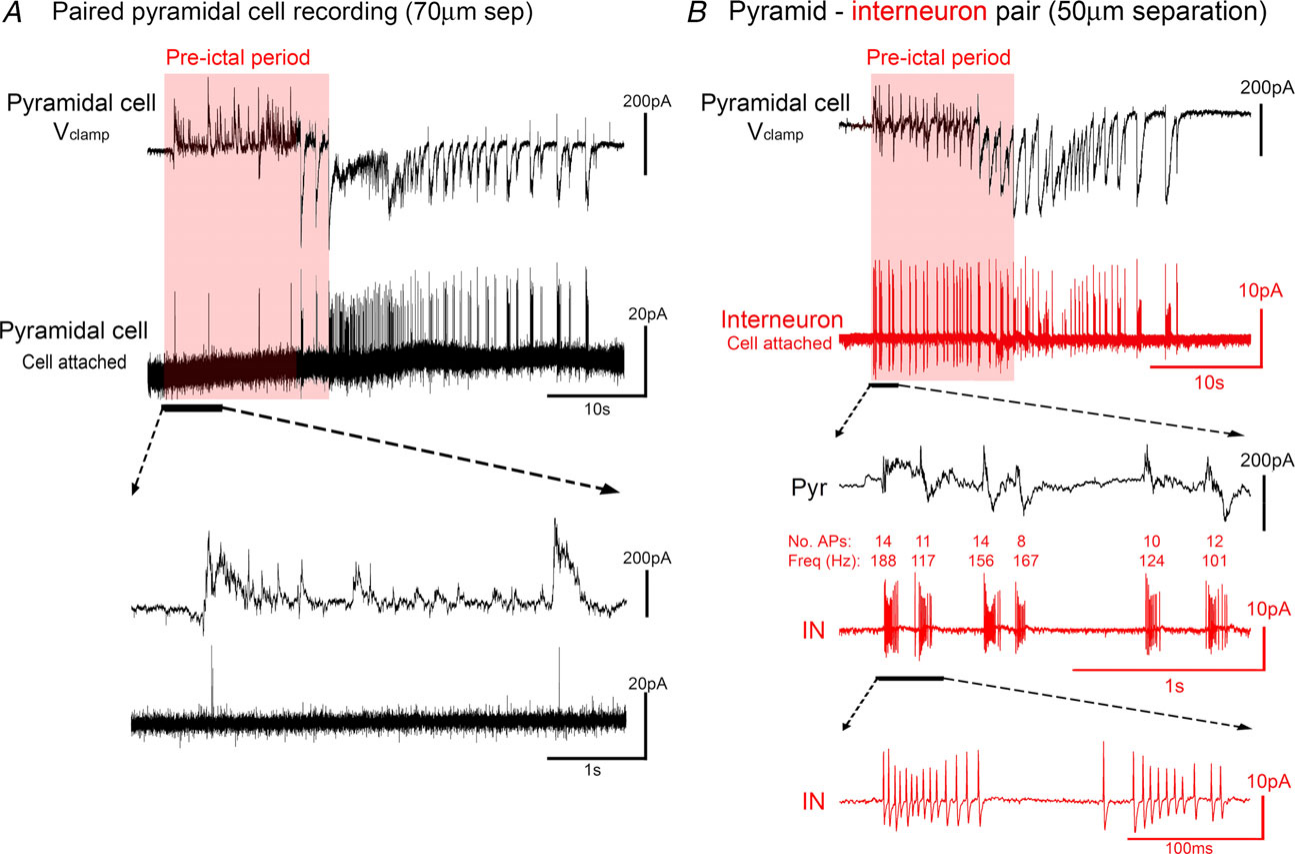
Differences in recruitment patterns between pyramidal cells and interneurons *A*, paired recording of two juxtaposed, layer 5 pyramidal neurons, with one recorded in *V*_clamp_ at −30 mV, and the other recorded in cell‐attached mode, using an electrode filled with ACSF, to record its firing patterns. Note the minimal firing in the pre‐ictal period. *B*, the same recording arrangement, except recording a non‐pyramidal somata with the cell‐attached electrode. Note the intense and sustained bursts of action potentials, at rates exceeding 200 Hz, from the very start of the pre‐ictal period. [Color figure can be viewed at wileyonlinelibrary.com]

**Figure 2:**
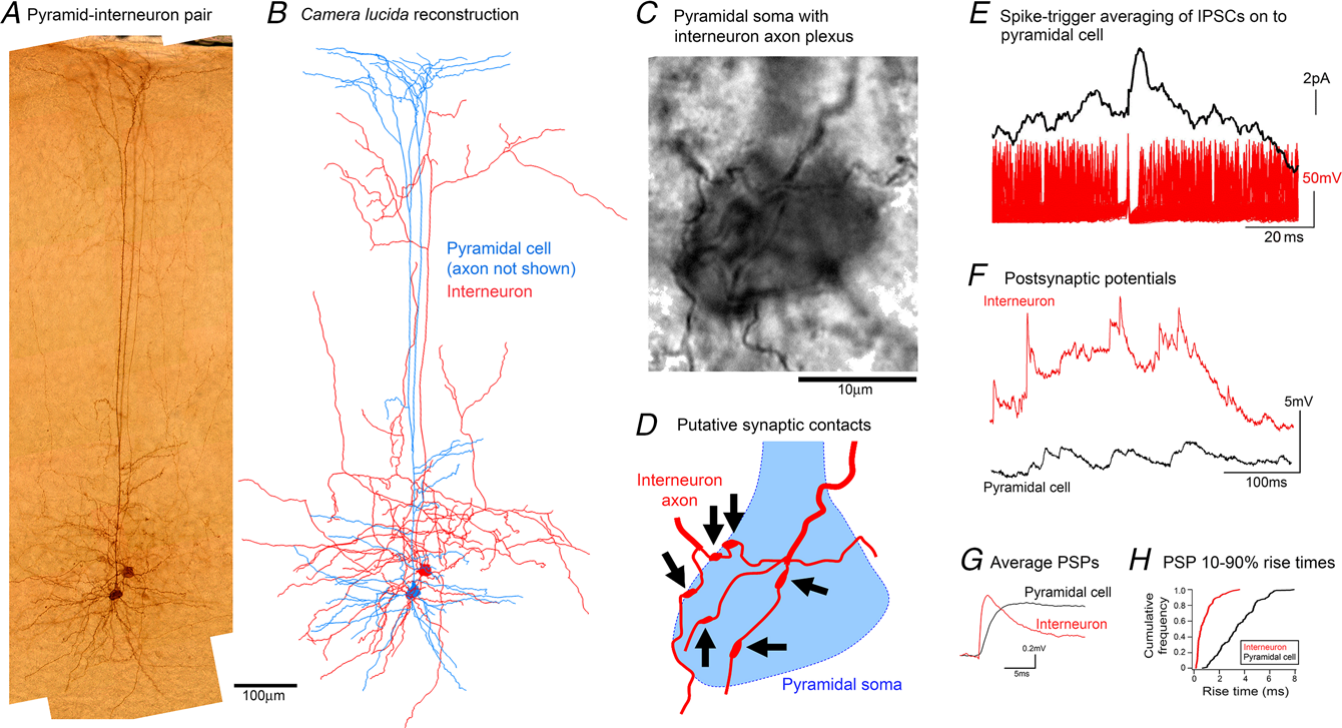
Rapid feedforward inhibition onto a pyramidal cell, mediated by a fast‐spiking interneuron *A* and *B*, photomicrograph (*A*) and camera lucida drawing (*B*) of a connected pyramidal cell–basket cell connected pair. The axonal morphology of the interneuron is consistent with descriptions of the type1 class of basket cells found in rodent visual cortex, as described by Sjöström and colleagues (Buchanan *et al*., 2012). *C*, high magnification view of the same section shown in *A*, showing the pyramidal soma enveloped by the interneuronal axonal plexus. *D*, schematic representation of *A* to show the locations of several putative interneuronal synaptic contacts onto the pyramidal soma. *E*, spike triggered averaging of the pyramidal voltage clamp recording, based on the timing of action potentials recorded in the cell‐attached interneuron, illustrating a short latency, inhibitory postsynaptic current. The example shows an almost instantaneous initial rise, that is too fast for the synaptic event, but is instead most probably caused by noise riding on top of the rapidly rising event. *F*, subsequent whole cell current clamp trace from the basket cell, and for comparison a similar trace from a layer 5 pyramidal cell (not synchronously recorded). Current clamp recordings were taken at resting membrane potential (*E*_m_), and since the GABAergic reversal potential (−59 mV) was close to *E*_m_ (mean interneuronal *E*_m_ = −60.8 ± 4.8 mV (±SEM; *n* = 4); mean pyramidal *E*_m_ = −70.9 ± 3.6 mV), we assume that the majority of events are excitatory, although we have no direct means of separating inhibitory and excitatory PSPs in these traces. Note the large amplitude events with extremely fast kinetics in the interneuronal recording. *G*, average PSPs (*n* = 200) for a stuttering cell and the pyramidal cell. *H*, cumulative frequency plot of the 10–90% rise times for the two cells.

We reasoned that these cells might be the source of these inhibitory postsynaptic currents (IPSCs), a view consistent with prior reports of early recruitment of fast‐spiking interneurons to ictal events (Kawaguchi, 2001; Timofeev *et al*. 2002; Ziburkus *et al*. 2006; Cammarota *et al*. 2013; Librizzi *et al*. 2017). We therefore conducted a series of paired recordings of closely apposed pyramidal cells and interneurons (Figs 1 and 2). Initial presumptive cell classification was based on the DIC visualization, into pyramidal *vs*. non‐pyramidal shaped somata. We used a voltage clamp recording of a layer 5 pyramidal cell, at −30 mV, to provide both the timing of the discharges and also some indication of the relative involvement of both inhibitory and excitatory synaptic drives. The second cell was recorded in cell‐attached mode, to capture, non‐invasively, its firing pattern. We reported previously the low firing rates of pyramidal cells during the earliest synaptic bombardment (Trevelyan *et al*. 2006), and we show another example here to illustrate this (Fig. 1 *A*). There are, however, other cells that are recruited (Fig. 1 *B*) during this pre‐ictal period. We made paired recordings, where we held a layer 5 pyramidal cell at −30mV and recorded a second cell in either current clamp or cell attached mode. Of these paired recordings, seven showed high firing rates during the period prior to pyramidal activation (‘pre‐ictal’). These cells showed extreme levels of firing, at 100–300 Hz, with the bursts time‐locked to the rhythmic bursts of synaptic bombardment of the co‐recorded pyramidal cell. These bursting neurons typically had non‐pyramidal somata. Remarkably, we were able to perform spike trigger averaging of the postsynaptic voltage clamp recording in the pyramidal cell during these intense synaptic bombardments, and isolate unitary inhibitory synaptic connections between the pairs of cells (5 out of 7 pairs; Fig. 2 *E*), with short‐latency kinetics typical of basket cells (Kraushaar and Jonas, 2000; Maccaferri *et al*. 2000). The fact that this could be achieved even with the level of synaptic noise from the concurrent synaptic bombardment is surprising, and indicative of a particularly strong local synaptic connection.

We then repatched the same neuron in whole cell mode for more extensive cellular characterization. If ‘interictal bursting’ interneurons do indeed provide the rapid feedforward inhibition, then they must be rapidly activated. A key factor is therefore likely to be the kinetics of their excitatory drive. To quantify this, we analysed the rise times of PSPs in four interneurons and seven layer 5 pyramidal cells (Fig. 2 *F* and *G*). PSP rise times in interictal bursts were significantly faster in the interneurons than in pyramidal cells (interneuronal range of median rise times = 0.56–1.52 s (*n* = 4); pyramidal range = 1.70–3.34 s (*n* = 7); *P* = 0.0061, Wilcoxon rank sum test; Fig. 2 *H*). Two factors may account for this difference: the faster kinetics of interneuronal glutamate receptors (Geiger *et al*. 1995), and excitatory synapses being closer to the soma in interneurons (Peters and Jones, 1984). In two cells, the fill of the axon was sufficiently good for us to identify axon connections onto pyramidal somata (Fig. 2 *A–D*). These recording thus provide an explanation of how the disynaptic inhibition of local interneurons during spreading ictal activity may be expedited to oppose the monosynaptic excitatory drive onto the pyramidal cells.

To facilitate searching for specific interneuron classes, we employed a genetic labelling strategy, by injecting a viral vector carrying the floxed tdTomato reporter gene into mouse pups carrying the CRE‐recombinase gene either under the PV or the SST promoters. This provided a cell‐class specific fluorescent label of either PV or SST interneurons. We then performed targeted patch clamp recordings in young adult animals (5–12 weeks old) to investigate whether the patterns of activation that we had recorded in adolescent animals was maintained into adulthood. We analysed the firing patterns of both PV and SST interneurons during a time period delineated by two readily identifiable features in the pyramidal *V*_clamp_ recordings, namely, the start of the rhythmic synaptic barrages (*T*_i_) and the time of the maximal inward current (*T*_e_), which our previous work had showed to correspond with the main recruitment of local pyramidal population (Trevelyan *et al*. 2006). We refer to this epoch (*T*_i_ to *T*_e_) as the pre‐ictal period.

**Figure 3:**
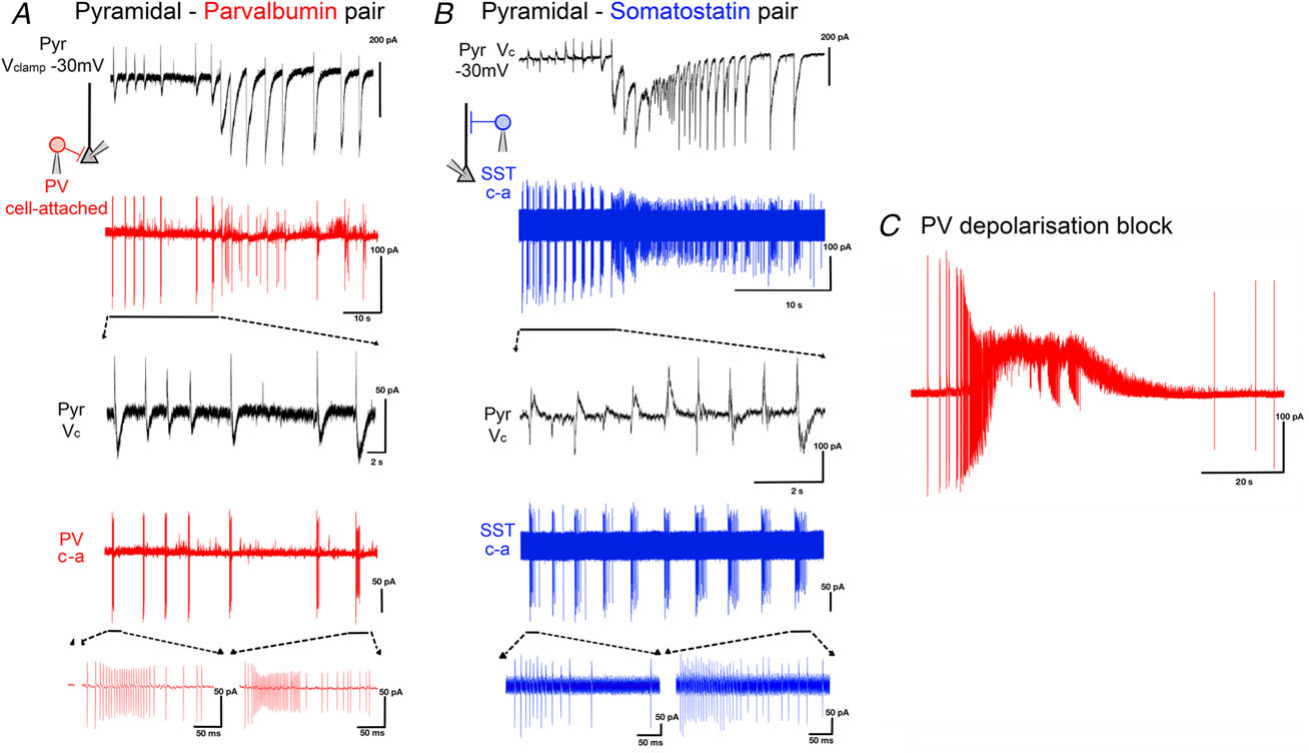
Intense rhythmic activation of both PV and SST interneurons during the pre‐ictal period *A*, paired recording of a pyramidal cell (black trace: *V*_clamp_ at −30mV) and a cell attached (‘c‐a’) recording of a PV interneuron (red trace) during a propagating ictal event. *B*, equivalent paired recording of a pyramidal cell and a SST interneuron (blue). *C*, example recording of PV interneuron, showing evidence of depolarizing block (extremely truncated APs, but which recover post‐ictally) during the ictal event. [Color figure can be viewed at wileyonlinelibrary.com]

**Figure 4:**
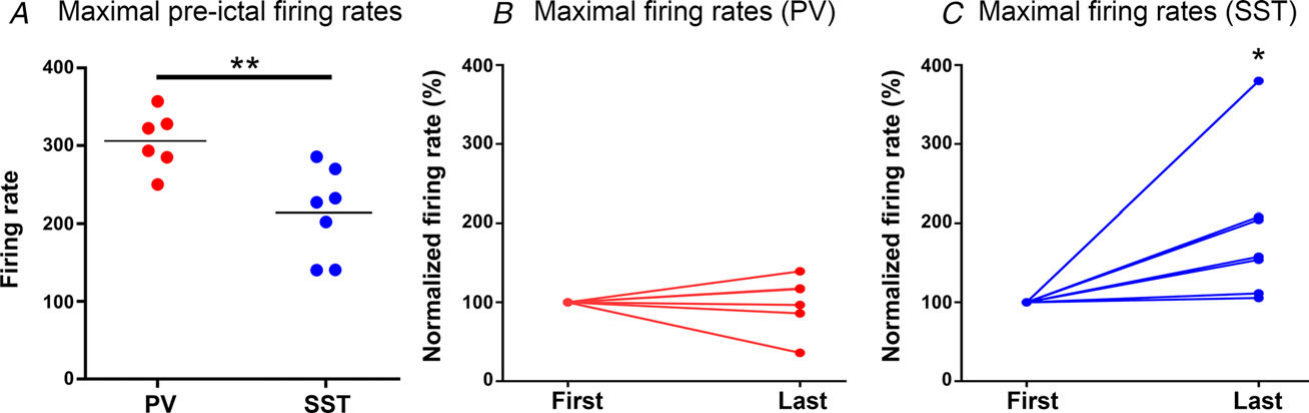
PV and SST interneurons display different pre‐ictal firing patterns *A*, population data from 13 neurons, recorded from 8 animals, showing that PV interneurons achieve significantly higher firing patterns (*P* = 0.0066, unpaired *t* test). *B*, PV firing does not change during the course of the pre‐ictal time period (*P* = 0.85, unpaired *t* test, analysis was performed on raw data), whereas SST firing (*C*) shows a marked, and highly significant, increase during this same period (*P* = 0.0082, unpaired *t* test, analysis was performed on raw data). [Color figure can be viewed at wileyonlinelibrary.com]

These recordings confirmed that PV interneurons displayed very intense firing during the pre‐ictal period (up to 350 Hz; average = 270 ± 45 Hz; *n* = 6) (Figs 3 *A* and *C* and 4 *A*). Of note, PV cells transitioned into a maximal firing rate from the first pre‐ictal event and maintained this rate throughout the pre‐ictal period (average = 262 ± 78 Hz; *n* = 6) (Fig. 4 *B*). SST cells were also active at this time, but showed some key differences from the PV firing pattern. SST interneurons fired at a much lower initial rate (average = 127 ± 41 Hz; *n* = 7), but this increased significantly over the course of this pre‐ictal period (average = 214 ± 52 Hz; *n* = 7; Fig. 4 *C*). Thus, these synaptic inhibitory barrages onto pyramidal cells arise from both the main classes of interneurons that target them.

Five out of six PV interneuron cell attached recordings showed abrupt termination of spiking just after the onset of the ictal event (Fig. 3 *C*), suggestive of depolarizing block (as described by others (Ziburkus *et al*. 2006), but because we were recording in cell attached mode, we cannot definitively state this). In contrast, all but one of the SST cells continued firing throughout the entire ictal event (Fig. 3 *B*).

**Figure 5:**
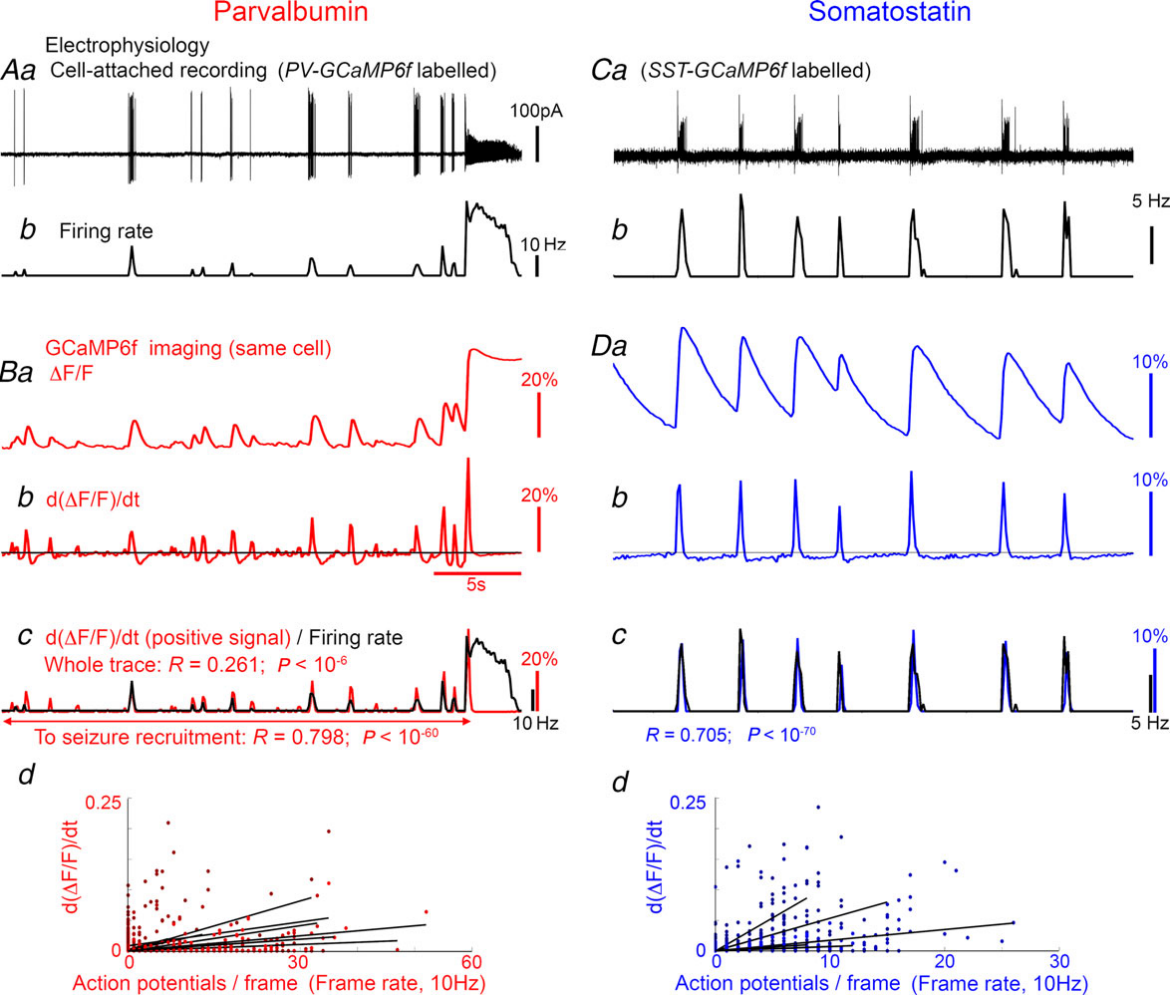
Non‐invasive Ca^2+^ imaging using GCaMP6f yields an accurate estimate of relative, but not absolute, firing rates in both PV and SST interneurons *Aa*, cell attached recording of a GCaMP6f‐labelled PV interneuron; *b*, the firing rate during each individual frame of the concurrent GCaMP6f imaging. *Ba*, Ca^2+^ imaging processing of the same cell, showing the Δ*F*/*F*_min_ signal; *b*, the derivative of this signal. The positive values of d(Δ*F*/*F*)d*t* (above the black line in *Bb*) superimposed on to the electrophysiologically measured firing rate (*Bc*, black). *Bd*, all recordings (14 recordings of 9 brain slices) showed strong correlation between the instantaneous AP count and the d(Δ*F*/*F*)d*t* signal, although there was considerable variation in the gradient (scaling factor). *C* and *D*, example recording and firing rate for an SST interneuron (*C*), and GCaMP6f signal of that neuron over the same time period (*D*). Again, there was large variation in the gradient for the correlation between firing rate and Ca^2+^ signal, meaning that the pattern of firing was accurately described by the GCaMP6f, but the precise firing rate cannot be derived uniquely from the Ca^2+^ signal (18 recordings of 6 brain slices).

**Figure 6:**
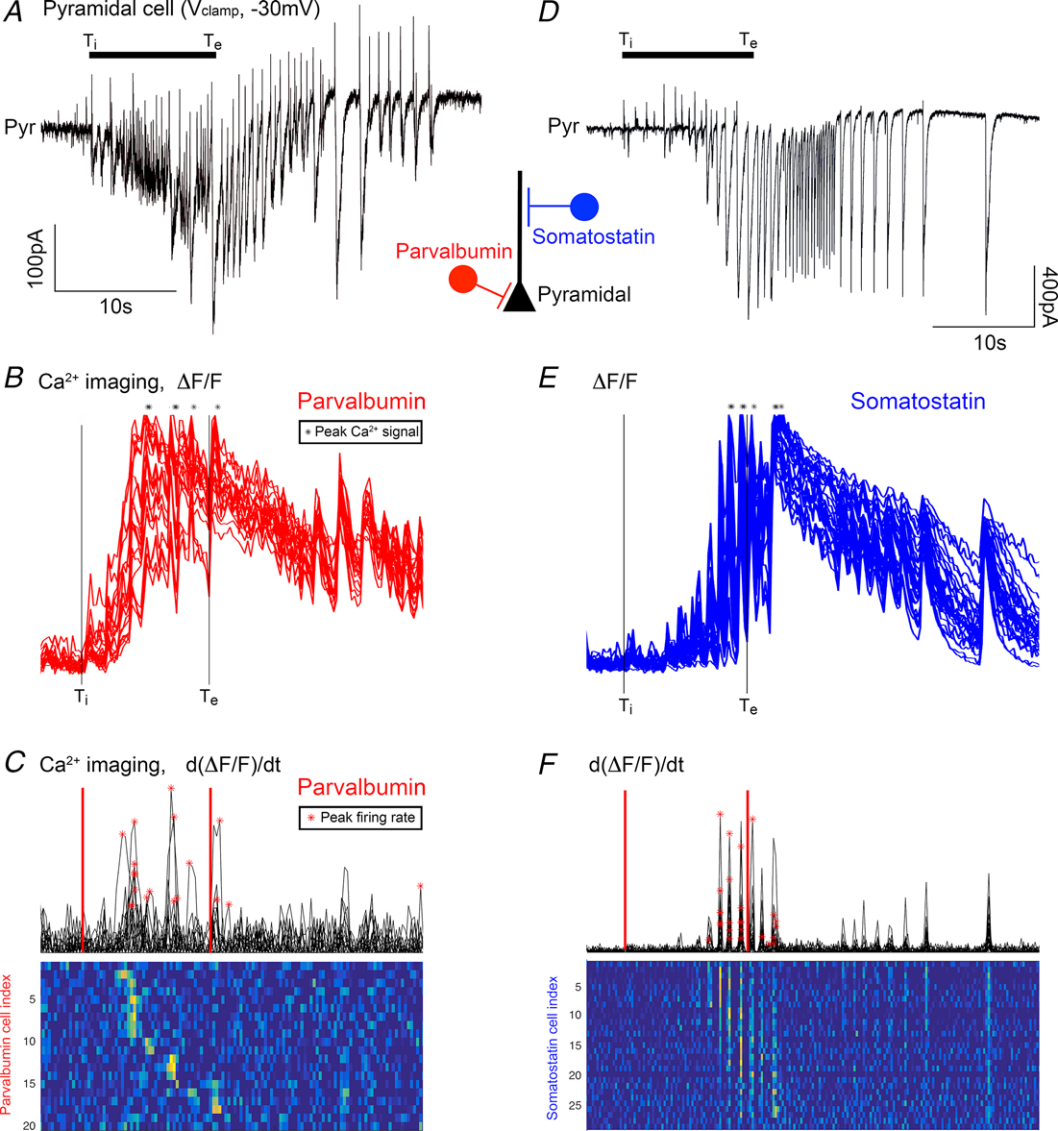
Example recordings of PV and SST interneurons during recruitment to a propagating ictal event *A* and *D*, voltage clamp recording of layer 5 pyramidal cells, held at −30mV, to illustrate the time course of the stages of recruitment to a propagating ictal event. The electrophysiology recordings are aligned with GCaMP6f imaging of local populations of either parvalbumin‐ (*B* and *C*) or somatostatin‐ (*E* and *F*) expressing interneurons. *B*, the Δ*F*/*F*_min_ signal, normalized to the peak height, for 21 neurons, within 150 μm of the recorded pyramidal neuron. The stars represent the time of the peak Ca^2+^ signal for each cell, during the entire ictal event. The vertical lines denote two easily, and objectively, identified time points from the pyramidal cell recording: *T*_i_, the start of the period of inhibitory restraint, as indicated by the onset of rhythmic synaptic bombardment in the pyramidal cell recording, and *T*_e_, the time of the peak excitatory current. *C*, the d(Δ*F*/*F*_min)_d*t* signal, with the times of peak firing rates indicated by the red asterisks. Note that these do not correspond to the time of the peak Ca^2+^ signal *B*. *E* and *F*, similar analyses of GCaMP6f imaging of SST interneurons.

**Figure 7:**
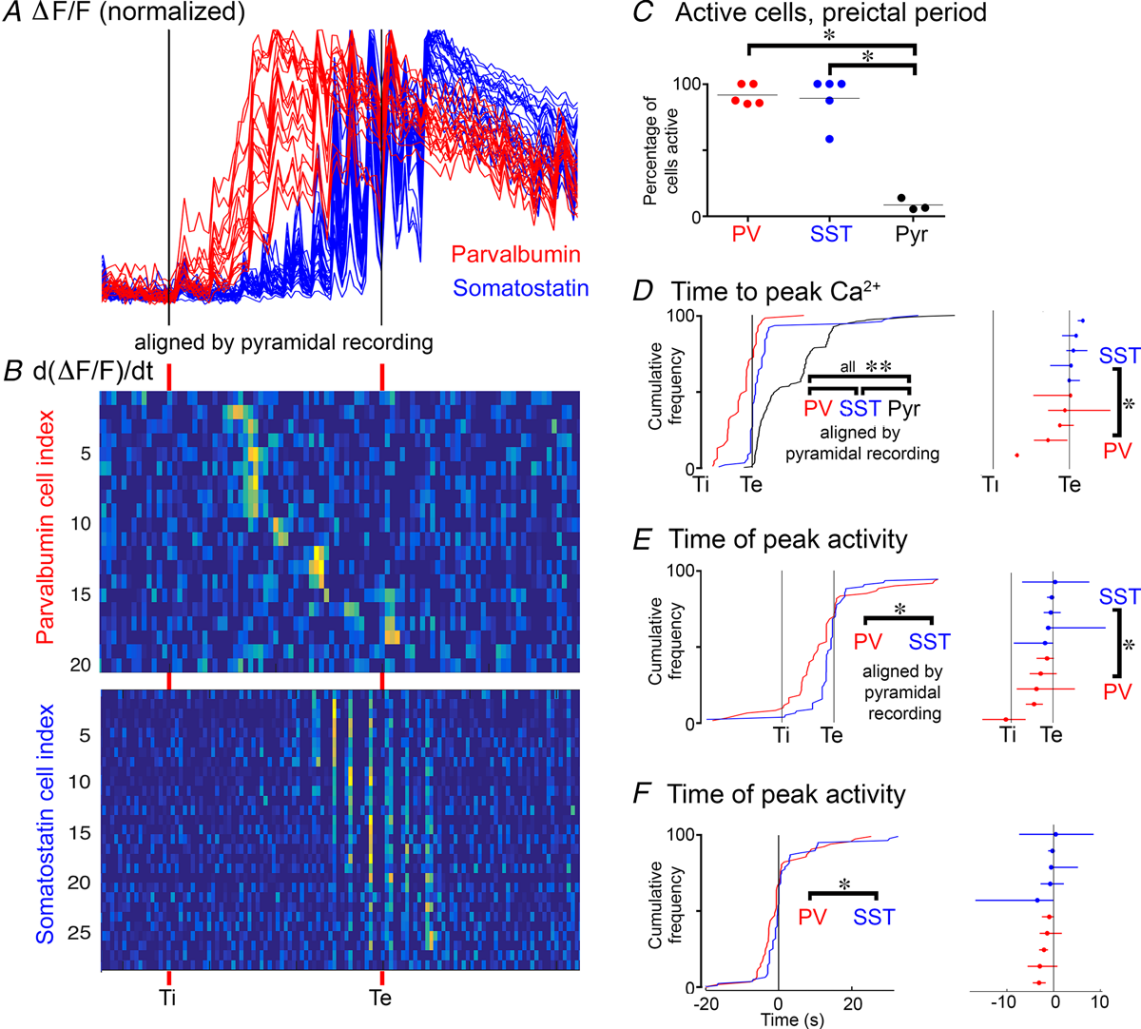
Sequential recruitment of PV and SST interneurons to a propagating ictal event *A*, superimposed views of the Δ*F*/*F*_min_ signals from the example shown in Fig. 6, aligned by *T*_i_ and *T*_e_. *B*, the estimate of firing rate, provided by d(Δ*F*/*F*_min)_d*t*, again aligned to *T*_i_ and *T*_e_. *C*, data from 13 adult mice, showing the proportion of the three cell classes activated in the pre‐ictal period; 92% of PV cells and 89% of SST cells show significant Ca^2+^ signal during this period, compared to just 15% of pyramidal cells (^*^PV *vs*. Pyr, *P* = 0.0001; SST *vs*. Pyr, *P* = 0.0001, ANOVA with Tukey's test). *D*, population data from 75 PV (5 mice), 80 SST interneurons (5 mice) and 204 pyramidal cells (4 brain slices, 3 mice), showing the distribution of times of peak Ca^2+^ signal during ictal events (^**^PV *vs*. SST, *P* = 0.003; PV *vs*. Pyr, *P* = 1.1 × 10^−12^; SST *vs*. Pyr, *P* = 1.2 × 10^−8^). The different data sets are aligned according to *T*_i_ and *T*_e_. The right panel shows the median and the 25–75 percentile range for all animals (PV *vs*. SST, *P* = 0.0159, Wilcoxon rank sum test). *E*, the distribution of peak firing rates for PV and SST interneurons, as estimated from the d(Δ*F*/*F*_min)_d*t* signal (^*^ *P* = 0.00012; unpaired *t* test). The right panel shows the median and the 25–75 percentile range for all animals (^*^ *P* = 0.0159; Wilcoxon rank sum test). *F*, the same data set plotted in real time, relative to *T*_e_ (*T*_e_ = 0 s; ^*^ *P* = 0.018). Note that by every metric, PV cells lead the SST population, which in turn, leads the pyramidal population (*T*_e_).

For a systematic investigation of large numbers of neurons, we employed an imaging strategy, using conditional expression of GCaMP6f in the three main neuronal classes (pyramidal neurons and PV and SST interneurons), which allowed us to garner data at a population level, while still providing cell class‐specific information (Figs 5, 6, 7). We made brain slices from young adult mice (5–12 weeks) expressing GCaMP6f under PV, SST or Emx1 promoters. Although these promoters can lack sensitivity, they show high specificity, meaning that while we may miss some neurons, we were confident regarding their grouping into the three most numerous neuronal cell classes.

One concern with these studies was that the intrinsic Ca^2+^ buffer, parvalbumin, might distort the GCaMP6f signal, which might prevent a direct comparison of imaging experiments in the PV and SST populations. To assess this issue, we conducted a series of cell‐attached recordings of the firing patterns of single cells in 0 Mg^2+^ ACSF (9 PV interneurons, Fig. 5 *A*; 7 SST interneurons, Fig. 5 *C*), while simultaneously imaging fluctuations in the GCaMP6f signal. GCaMP6f shows a rapid increase in fluorescence coincident with the sudden influx of Ca^2+^, followed by a slow decay. When PV cells entered episodes of depolarization block, the Ca^2+^ signal appeared to peak, and did not show further increases thereafter, during the ongoing seizure activity (Fig. 5 *Ba*). Prior to this, though, the firing pattern was well described by the positive values of the first derivative of the Ca^2+^ signal (d(D*F*/*F*)/d*t*; Fig. 5 *Bc*). Notably, the scaling of the Ca^2+^ signal to the firing rate varied between preparations substantially, and consequently, we were unable to use the Ca^2+^ signal to estimate the precise firing rate, but the general pattern of firing over the course of the movie was very well represented for both cell classes. Importantly, the PV expression did not confer any apparent effect on the GCaMP6f signal.

We imaged local populations of labelled neurons adjacent to a pyramidal cell being recorded in voltage clamp at −30mV (imaged cells <200 μm from the recorded neuron; Fig. 6). As in Figs 1 and 3, the electrophysiological recording was used to denote the precise timing of the different stages of the propagating ictal event, and specifically the timing of the pre‐ictal period of inhibitory restraint.

We found that all three cell classes were active during the pre‐ictal period (Figs 6 and 7), albeit, to varying degrees. While almost all of the PV‐GCaMP6f labelled interneurons (92%) were active during the pre‐ictal period, only a small percentage of pyramidal cells were active then (15%) (Fig. 7 *C*). Consistent with our prior electrophysiological recordings, almost all of the SST interneurons were also active during the pre‐ictal period (89%), equal to the participation of the PV interneurons (Fig. 7 *C*). Notably, however, further analysis of the Ca^2+^ signal showed a consistent pattern of PV activity leading the other cell classes (Figs 7 *D–F*). In particular, the timing of both the peak Ca^2+^ (Fig. 7 *D*) and also the peak activity in the PV population (Fig. 7 *E*) was significantly in advance of the peak activity in the SST population. This sequential recruitment recapitulates the pattern of physiological activation of these interneuronal populations, in response to an afferent glutamatergic drive, where one sees a feedforward inhibition that is initially targeted towards the soma (the presumptive PV population), but then shifts towards the dendrites (the presumptive SST population) (Pouille and Scanziani, 2004).

### Interictal activity patterns

**Figure 8:**
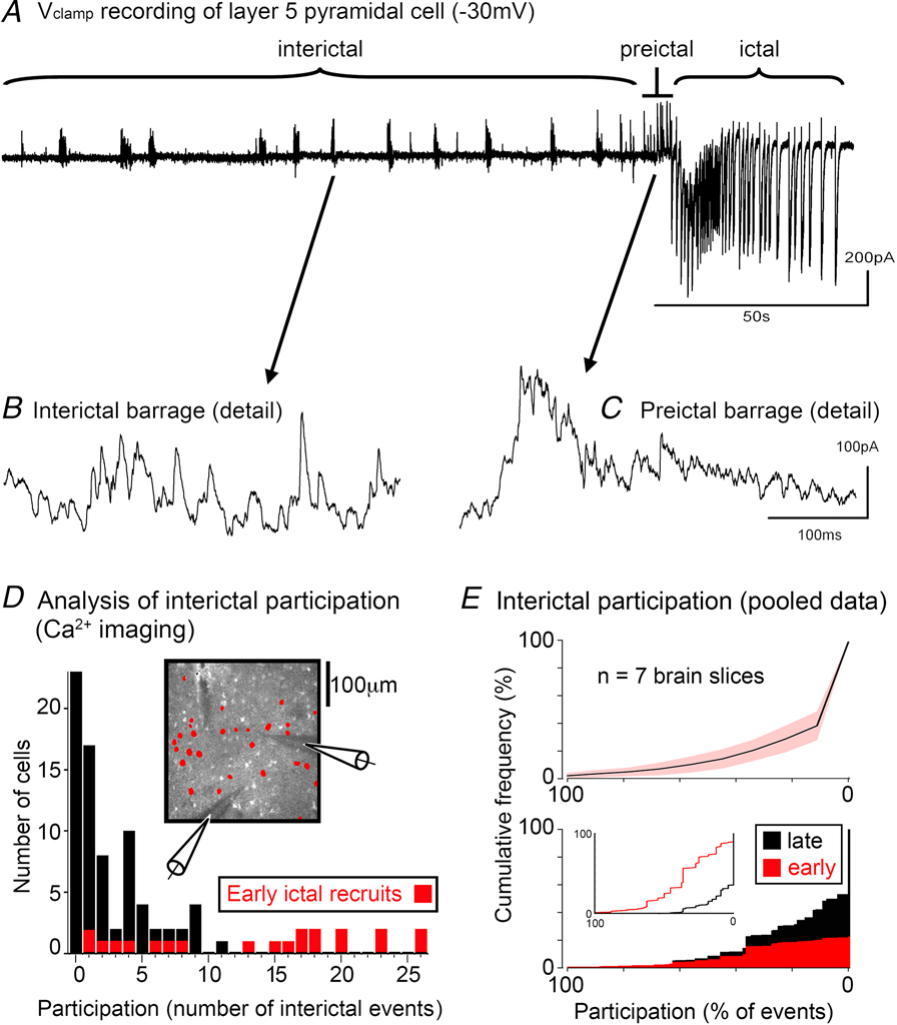
Similar activity profiles during interictal events and the pre‐ictal period *A*, an extended *V*_clamp_ recording of a layer 5 pyramidal cell held at −30 mV, showing repeated interictal events, ultimately leading into a full ictal event. *B*, expanded view of an interictal event showing the high frequency barrage of IPSCs. *C*, similar high frequency IPSC barrages are also seen in the pre‐ictal period. *D*, frequency histogram of neuronal cell participation in interictal events (OGB1 labelling). The timing of events is determined from the IPSC discharges in the synchronous *V*_clamp_ recording of pyramidal cells. All the analysed neurons showed significant Ca^2+^ signals during a full ictal event (astrocytes were identified using the astrocyte‐specific dye, SR101 (Nimmerjahn *et al*. 2004), and then excluded from the analysis). The labelling in these experiments does not distinguish between the different neuronal classes. Analysing only those neurons showing a significant Ca^2+^ ictal signal, the histogram plot shows, for every neuron, the proportion of interictal events that cell participated in. The cells recruited to the full ictal event at the first network crisis (cf. Fig. 1) are coloured red, whereas the late recruits are coloured black. This colour coding shows that all the core participants of the interictal events are included in this group of early ictal recruits. *E*, upper panel, the cumulative frequency plot, ordered from participation in every interictal event (100%) to a complete absence of participation (0%), in 7 brain slices (966 cells; mean = 138 cells/slice; range = 36–251 cells/slice; number of interictal events/slice, mean = 11.6; range 7–26). Note that for every slice, the median value was only surpassed for the final group (‘no participation’). In other words, the majority of neurons are not active during interictal events. Lower panel, cumulative frequency plot showing the interictal participation profile of all cells pooled together (black), and the subpopulation that are active during the pre‐ictal period (red). The inset shows the same data, but replotted separately for the early (pre‐ictal) and the late recruits.

**Figure 9:**
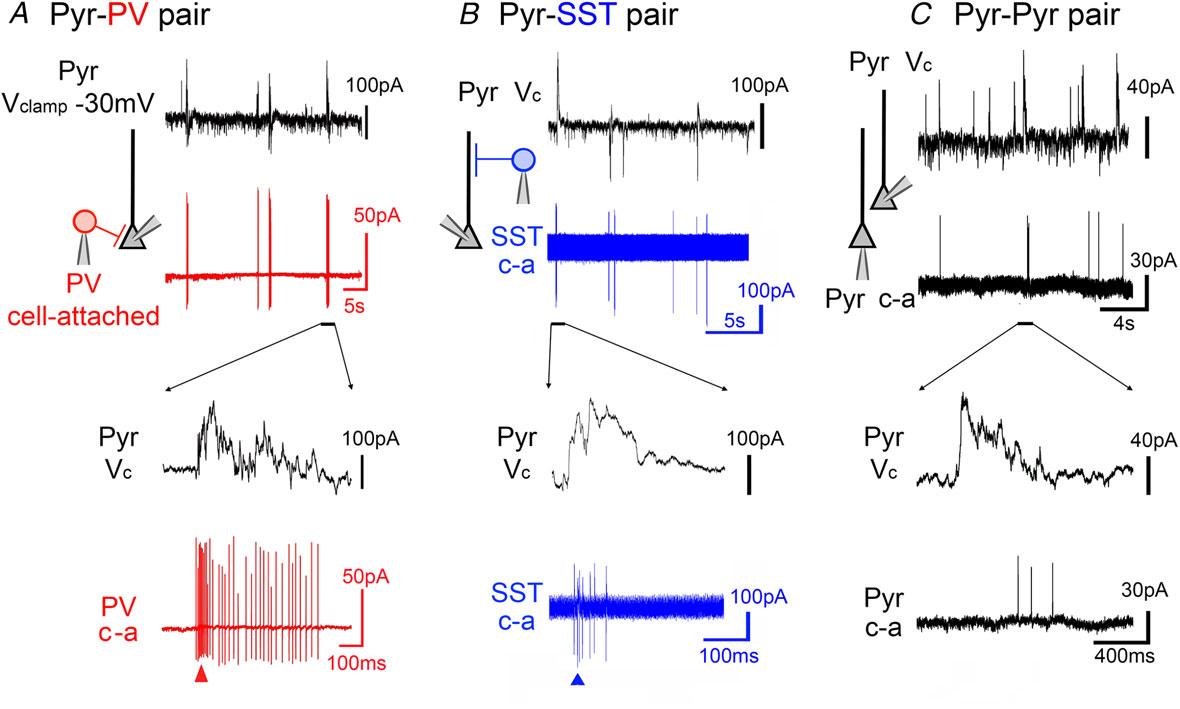
Interictal events are associated with intense interneuronal firing *A*, example paired recordings illustrating the firing patterns during interictal events of a parvalbumin‐positive interneurons (red, cell attached recording, ‘c‐a’). *B* and *C*, somatostatin‐positive interneuron (blue) (*B*) and a pyramidal cell (*C*) (lower traces). In each case, an enlarged view is shown below. [Color figure can be viewed at wileyonlinelibrary.com]

**Figure 10:**
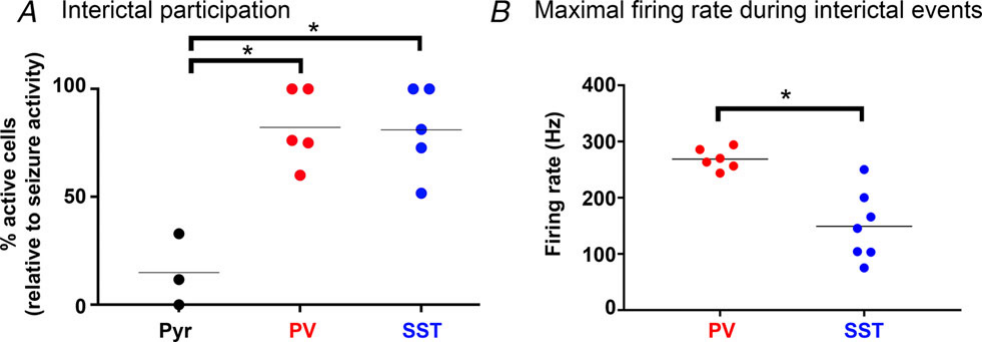
Interneurons participate in interictal events more intensely than pyramidal cells *A*, significantly larger proportion of PV (82%) and SST (81%) interneurons are active during interictal events than pyramidal cells (14.9%) (^*^PV *vs*. Pyr, *P* = 0.0014; SST *vs*. Pyr, *P* = 0.0016, ANOVA with Tukey's test). *B*, firing rates are typically higher in PV than in SST interneurons (^*^ *P* = 0.0023, Wilcoxon rank sum test) [Color figure can be viewed at wileyonlinelibrary.com]

Our patch clamp data suggested that interictal events (Figs 8, 9, 10) had close parallels to the patterns of activity seen during the pre‐ictal periods. These events, which last about a second, were never recorded in normal ACSF, but developed within minutes after the washout of Mg^2+^. Thereafter, they occurred regularly (mean interval ∼20 s) for the periods between the first few ictal events. Notably though, after the first few full ictal events, both the interictal events and also the periods of restraint prior to each ictal event ceased to occur, seemingly in parallel. We analysed participation in interictal events up to the third full ictal event. Both pre‐ictal and interictal events were characterized by high frequency, large amplitude IPSCs onto pyramidal cells which depressed during the barrage (Fig. 8 *A–C*).

As with the pattern of activity during the pre‐ictal period, Ca^2+^ network imaging revealed that the participation in interictal events was similarly skewed (Fig. 8 *D* and *E*). Participation was generally rather low in these events (113 interictal events; mean participation = 16.0%), but notably, the neurons that were active during the pre‐ictal period were also found to be those most likely to participate in interictal events (Fig. 8 *D* and *E*). For all seven brain slices (6 different animals), histograms of the participation levels in interictal events show that the modal group is always the ‘no participation’ group. Cumulative frequency plots, again for all slices, counting up from those cells participating in the most interictal events, show that the median also was this ‘no participation’ group. Thus, only a minority of neurons appear to be active during interictal events. There was a highly significant difference in the level of participation between those neurons that were active in the pre‐ictal period *vs*. those that were recruited only in the later stages of the ictal event (‘pre’, median = 37% participation; ‘late’, median = 0% participation; Wilcoxon rank sum test, *P* = 5.6 × 10^−8^).

Analysis of mice with cell class‐specific GCaMP6f expression also showed high levels of both PV (65 out of 79 cells (82%); 52 events imaged in 5 brain slices) and SST (67 out of 83 cells (81%); 60 events, 5 slices) interneuron involvement, and low levels of pyramidal activity (31 of 206 cells (15%); 30 events, 3 slices; Fig 10 *A*). This was further confirmed electrophysiologically, using cell‐attached recordings of fluorescently identified interneurons. PV cells fired during every interictal event (*n* = 79 events; 6 slices), with an average of 8.5 action potentials (range = 3–36). PV interneurons showed remarkable transitions, from complete quiescence to extremely high frequency firing, at times achieving an almost instantaneous switch to 300 Hz firing during the interictal events (Figs 9 *A* and 10 *B*). SST interneurons also showed intense activation (up to 250 Hz), although at a significantly lower level than for PV interneurons (Figs 9 *B* and 10 *B*). Similar to the PV interneurons, SST interneurons also fired during every interictal event (81 events, 7 slices; average of 10.4 action potentials; range = 3–37 action potentials). SST activity was marginally higher than PV activity during interictal events (*P* = 0.0630, Wilcoxon rank sum test).

Finally, we performed paired recordings of pyramidal cells (Fig. 9 *C*) during the interictal events. In stark contrast to the PV and SST populations, pyramidal cells experienced large depolarizing shifts during interictal events, but fired only intermittently (8 cells from 8 slices; 138 events; average activity = 1.5 action potentials per event (range = 0 to 9); proportion of quiescent events = 39.0 ± 8.6%). Thus, the activity of all three cell classes was very similar during interictal events and in the immediate pre‐ictal periods. Notably, we previously showed, by holding pyramidal cells in voltage clamp close to *E*_GABA_, that the glutamatergic drive at these times is very strong, just like the pre‐ictal period (Trevelyan *et al*. 2006; Parrish *et al*. 2018 *a*). We conclude therefore that the interictal events in this model of epileptic activity should be regarded as equivalent network events to those occurring during the pre‐ictal period, and both represent instances of ‘network crisis’, when there is intense glutamatergic drive and yet the pyramidal activity is far lower than might be expected on account of the restraining function of the local interneuronal populations.

## Discussion

The study of epileptiform discharges offers many insights into the design of cortical networks (Trevelyan, 2016). The zero Mg^2+^ *in vitro* model remains a mainstay of epilepsy research because of the insights it provides into the nature of the interneuronal response to surges of network activity. Even though this model does not capture all facets of the chronic epileptic condition, the pattern of activity during propagation away from the ictal focus in human seizures appears to follow very similar patterns (Schevon *et al*. 2012). Here we used it to clarify what the involvement is of the two most populous interneuron types in these events. We examined both the immediate pre‐ictal period (immediately before the pyramidal recruitment) and interictal events. These appear to be very similar events, although they appear slightly more intense in the pre‐ictal bursts. The key features are that there is a substantial glutamatergic drive, albeit with little local pyramidal activity, but very intense burst firing in both PV and SST interneurons. The source of the glutamatergic drive is relatively clear in the pre‐ictal period, since the glutamatergic drive correlates well with bursting activity in adjacent territories that are already incorporated into the ictal event (Trevelyan *et al*. 2006). The source is less easily identified for the interictal bursts, although paired recordings from distant sites, or fast, wide‐field, Ca^2+^ network imaging shows that these often propagate at speed across the network. On other occasions, though, these events are quite focal, and possibly triggered by glio‐transmission (Fellin *et al*. 2004; Tian *et al*. 2005).

Perhaps the most notable finding was the intensity of SST firing during these events. SST interneurons were found regularly to fire in excess of 100 Hz, and at times at almost 300 Hz. PV interneurons fire at even higher rates, although this was more expected, given prior data regarding their involvement (Cammarota *et al*. 2013; Sessolo *et al*. 2015), their known high firing rate, and also the fact that they target the proximal dendrites and somata of pyramidal cells, consistent with the requirement for a fast and powerful inhibition at these times. The extraordinarily high firing rates that we report here were recorded in cell‐attached mode, driven by synaptic inputs, suggesting that the more conventional experimental assays of firing rate, derived from current injections at the soma, through whole cell recording electrodes, may underestimate a neurons maximal potential firing rate.

Interestingly, the activity of the interneuron classes appears to be coordinated, both to the timing of the glutamatergic bursts on the pyramidal cells via collateral branches from the afferent input, but also with each other. Analysis of excitatory postsynaptic potentials in pyramidal cells and interneurons indicate that the glutamatergic input appears to be directed close to the somata of the interneurons, and at electrotonically more distal sites in pyramidal cells, facilitating a faster response in the interneurons, and thus explaining how a disynaptic feedforward inhibition could supersede a monosynaptic excitation on the pyramidal cells.

During the pre‐ictal period, both interneuron classes are activated from the start of the synaptic bombardment, but PV cells show their peak Ca^2+^ signal significantly in advance of SST interneurons, recapitulating what has been reported previously during physiological activation (Pouille and Scanziani, 2004). The majority of PV cell‐attached recordings show evidence of depolarization block (greatly reduced AP amplitude and occasionally a prominent baseline recovery, both of which recover, post‐ictally), and notably, this coincided with the peak Ca^2+^ signal. We also showed that the pattern of firing was reproduced remarkably accurately by the first derivative of the Ca^2+^ signal, although the scalar relationship to the AP train varied between experiments, preventing us from using the network imaging data to derive precise firing rates. These recordings, therefore, showed when a cell showed the most intense bursts, and this metric also showed that the PV activity significantly preceded the SST activity, both when normalized to the epoch specified by the pyramidal recording (Fig. 7 *E*), and also in real time (Fig. 7 *F*). This is further supported by our cell‐attached recordings, and is consistent with a recent report of an acceleration of PV activation ahead of SST and pyramidal cell activation, in two acute, pharmacological *in vivo* models (Miri *et al*. 2018).

Notably, none of our recordings of SST neurons showed evidence of depolarizing block. While depolarizing block of PV interneurons has been reported previously, this has generally been when bathed in 4‐aminopyridine, which blocks the voltage‐gated K^+^ channels (Ziburkus *et al*. 2006; Cammarota *et al*. 2013, although note the example in the Cammarota study, in 0 Mg^2+^). It is therefore helpful to demonstrate the same phenomenon arising in a different model where the ability of these neurons to repolarize has not been pharmacologically compromised. Why SST interneurons appear able to resist going into depolarizing block is not clear. One interesting association, though, is between the different firing patterns in SST and PV interneurons and their synaptic output. PV interneurons show a strongly depressing output, and so perhaps the cessation of PV firing aligns with when the firing output is functionally diminished. SST interneurons, on the other hand tend to show a facilitating output, which is mirrored also in the progressive increase in firing during the pre‐ictal period. Similar activity has also been described for more physiological levels of activation of these two interneuronal classes (Pouille and Scanziani, 2004).

The idea, that the coordinated activity of both PV and SST interneurons might be required to achieve a full veto of pyramidal activity, extends our understanding of the restraint mechanism in important ways. The asymmetric arrangement of synapses on the dendritic trees of pyramidal cells, with the excitatory drive being located distally, and the existence of a very dense proximal cluster of inhibitory synapses, clearly facilitates a strong inhibitory control over their firing (Trevelyan, 2016). Since this proximal inhibition derives largely from PV interneurons, these had been presumed to be the essential component of the restraining interneuronal response. Furthermore, the inhibitory synaptic barrage is clearly very high frequency, which was thought to reflect the uniquely high firing rates shown by this class of interneuron. Our recordings show now that the SST interneurons can also fire at a surprisingly high rate when activated naturally, through synaptic drive.

There are, also, several reasons for thinking that the PV output cannot provide the restraint on its own. First, would the rapidly depressing output of PV interneurons be able to provide a restraint that outlasted the excitatory drive? Second, PV interneurons, through their tightly synchronized firing, their direct action close to the pyramidal axon initial segment, and the fast kinetics of their synaptic output, create a very powerful synchronizing drive on pyramidal cells (Cobb *et al*. 1995). It is possible that the window of opportunity for pyramidal firing is squeezed out, at the kinds of firing rates we report here (200–330 Hz). Third, pyramidal cells have excitable dendrites, which sustain plateau potentials, which can dramatically increase the output of pyramidal cells by triggering short bursts of action potentials (Larkum *et al*. 1999). Fourth, the intense high frequency inhibition coincident with a large depolarizing drive will cause a very rapid rise in Cl^−^ levels in the pyramidal cells (Ellender *et al*. 2014), thereby potentially exacerbating the epileptic activity rather than restraining it (Bernard *et al*. 2000; Huberfeld *et al*. 2007; de Curtis and Avoli, 2016). Notably, for each issue, the SST population provides a complementary solution. First, SST interneurons appear to show a complementary pattern of firing and synaptic facilitation to the depressing output of PV interneurons, suggesting that there is a natural transition from proximally targeted, to distally targeted inhibition (Pouille and Scanziani, 2004). Second, the distal location of the SST synapses on the pyramidal dendrites will filter their effect at the pyramidal somata, even though we now know the SST interneurons fire at similarly high rates to the PV population. This low‐pass filtering of the SST input by the pyramidal dendrites should reduce the potential for synchronized, rebound spiking. Third, the shift in dendritic location of the dominant inhibitory drive may be predicated on a dangerous chloride‐loading of the pyramidal somata and proximal dendrites. And finally, the SST interneurons appear to provide a very effective suppression of dendritic excitability (Lovett‐Barron *et al*. 2012). The coordination of these two key interneuronal populations is likely brought about through specific features of the microcircuitry, involving (1) VIP‐expressing interneurons that target both populations although not equivalently, (2) gap‐junction coupling between interneurons of the same class (Galarreta and Hestrin, 1999; Gibson *et al*. 1999), which creates a cell‐class‐autonomous excitation, and (3) mutual inhibition of PV and SST interneurons. Note though that there may be brain area differences in this mutual inhibition, with the PV to SST connection apparently dominant in CA1 (Lovett‐Barron *et al*. 2012), whereas the SST to PV connection being more substantive in neocortex (Pfeffer *et al*. 2013). Finally, this view, of a far more significant role for SST interneurons in the inhibitory restraint, is consistent with recent work detailing their role in lateral inhibition in sensory processing, in both visual cortex (Adesnik *et al*. 2012) and somatosensory cortex (Veit *et al*. 2017).

**Figure 11:**
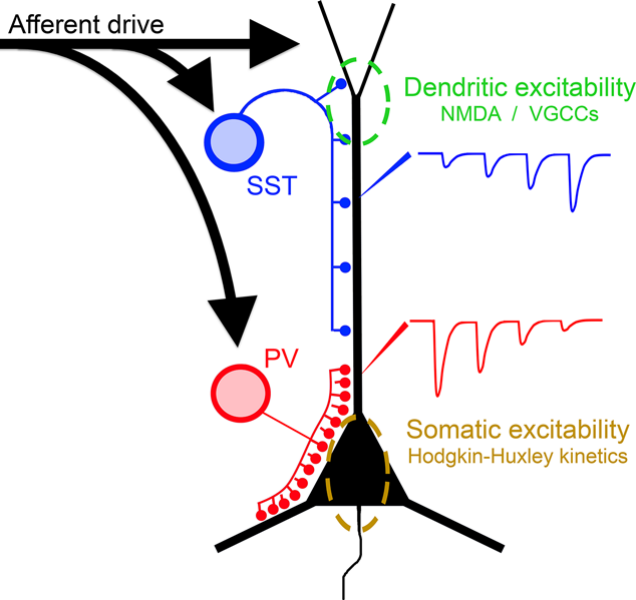
Schematic figure illustrating how the two interneuronal populations target different regions of excitability in pyramidal neurons Feedforward inhibition is provided by both PV and SST interneurons, but possessing different kinetics and targeted to different parts of the dendritic tree. The progressively depressing PV output targets the proximal dendrites, somata of pyramidal cells and axon initial segment of pyramidal cells, where there is a high density of Hodgkin–Huxley Na^+^ channels. In contrast, the peripheral dendrites, targeted by the SST interneurons, can sustain plateau potentials mediated by NMDA receptors and voltage‐gated Ca^2+^ channels. Dendritic potentials are a powerful drive for bursting behaviour in pyramidal cells. A vetoing inhibition of the pyramidal cells may require the coordinated inhibitory targeting of both these territories.

The model we propose for the rapid interneuron restraining response is shown in Fig. 11. Direct excitatory drive hits the pyramidal cells and both interneuronal classes. This creates a ‘front‐loaded’ inhibition, through the PV population onto the pyramidal cell somata, which is time‐locked to the glutamatergic bursts, by dint of the fact that interneuronal firing is driven by collaterals from the same axonal input. This PV effect, though, rapidly depresses, but over the same time period, the SST output increases, and is aimed at preventing the slower kinetic plateau potentials that underlie pyramidal bursting behaviour.

We are thus arriving at a more nuanced view of how the precise pattern of cortical microcircuitry can regulate the flow of activity in cortical networks. The rapid interneuronal response appears to be a coordinated effect of both PV and SST inhibition. On occasions, this clearly fails to restrain the network, which is when we see seizures. There has been much interest in trying to treat epilepsy by creating artificial inhibitory restraints, but we will need to take account of the subtle features of the endogenous mechanisms, including the precision of its timing and the interplay between the different inhibitory components, and this may not be trivial.

## Additional information

### Competing interests

The authors declare that they have no competing financial interests.

### Author contributes

Experiments were performed by N.K.C., R.R.P., C.M.G.S. and A.J.T. Data analysis were performed by N.K.C., R.R.P. and A.J.T. Experiments were conceived by N.K.C., R.R.P. and A.J.T. Manuscript was written by R.R.P. and A.J.T. All authors have read and approved the final version of this manuscript and agree to be accountable for all aspects of the work in ensuring that questions related to the accuracy or integrity of any part of the work are appropriately investigated and resolved. All persons designated as authors qualify for authorship, and all those who qualify for authorship are listed.

### Funding

This work was supported by Epilepsy Research UK and the Medical Research Council (UK).
